# Supplementary material for: A complex ePrescribing-based Anti-Microbial Stewardship (ePAMS+) intervention for hospitals combining technological and behavioural components: protocol for a feasibility trial
Source: Pilot Feasibility Stud. 2023 Jan 28;9:18. doi: 10.1186/s40814-022-01230-w (PMC9883604; doi:10.1186/s40814-022-01230-w)
Supplement: Supplementary file 3 — Additional file 3. Healthcare professional interview topic guide. [file 40814_2022_1230_MOESM3_ESM.docx]

**Topic guide Healthcare Professionals and Patient focus groups**

**Welcome & ground rules**

The purpose of the group today is to discuss your experiences/opinions of ePAMS+.

What you say is confidential and whatever views you express will be anonymised so that no one outside of this room will know who has said what.

You should therefore treat information and views expressed in this room as confidential.

Please feel free to say what you want and feel free to discuss comments or add your own experiences.

Please allow others to have their say (even if you disagree), ‘disagree without being disagreeable’, please respect other peoples’ viewpoints and try to allow quieter people some space to give their thoughts too.

With your consent, the discussions will be recorded so that we can analyse them later, but the data will be anonymised.

It’s not so much a question and answer session as a forum for discussion.

I may need to stop a discussion if going over time in order to get all topics discussed.

**Group introductions**

Researcher and participants (names and professions)

Record names/positions

State the key topics to keep the structure of the discussion

**Key themes for discussion (will include follow up questions the focus group facilitator can ask to explore the different components of ePAMS+)**

TOPIC 1 – experiences and opinions of ePAMS+

How would you describe your experiences of being involved with the intervention? (Positive and negative)

TOPIC 2 – potential wider usability of ePAMS+

In your opinion, how can we change the intervention to make it easier to use in other settings? How might this affect patient care?

**Closure**

Recap of key discussion points and explanation that the results will be written up and sent to participants for comments

Anything else to add?
