# Supplementary material for: A complex ePrescribing-based Anti-Microbial Stewardship (ePAMS+) intervention for hospitals combining technological and behavioural components: protocol for a feasibility trial
Source: Pilot Feasibility Stud. 2023 Jan 28;9:18. doi: 10.1186/s40814-022-01230-w (PMC9883604; doi:10.1186/s40814-022-01230-w)
Supplement: Supplementary file 4 — Additional file 4. Statistical analysis plan. [file 40814_2022_1230_MOESM4_ESM.docx]

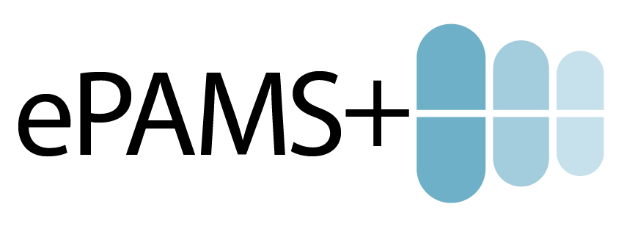


**Statistical Analysis Plan**

**CONFIDENTIAL**

| **Version No** | 1.0 |
| --- | --- |
| **Date Finalised** | 9 September 2022 |
| **Author(s)** | Imad Adamestam, Professor Christopher Weir |
| **CI Name** | Professor Aziz Sheikh |
| **CI Email address** | Aziz.Sheikh@ed.ac.uk |

The study is funded by National Institute for Health Research (NIHR) RP-PG-0617-20009. The funding reference number is ‘RP-PG-0617-20009’ and the project registration number is ‘ISRCTN13429325’.

| Signatures | |
| --- | --- |
| **Trial Statistician:** Professor Christopher Weir | **Date:** |
| **Chief Investigator:** Professor Sir Aziz Sheikh | **Date:** |

**Table of Contents**

[List of Abbreviations 3](#_Toc113611626)

[1. Introduction 4](#_Toc113611627)

[1.1 Background 4](#_Toc113611628)

[1.2 Objectives 5](#_Toc113611629)

[2. Statistical analysis section from the protocol 5](#_Toc113611630)

[*2.1 SAMPLE SIZE CALCULATION* 5](#_Toc113611631)

[*2.2 PROPOSED ANALYSES* 5](#_Toc113611632)

[3. Overall Statistical Principles 6](#_Toc113611633)

[3.1 Multiplicity 6](#_Toc113611634)

[4. Outcomes 6](#_Toc113611635)

[4.1 Primary outcome 6](#_Toc113611636)

[4.2 Secondary outcomes 7](#_Toc113611637)

[4.2.1 Antibiotic Prescribing 7](#_Toc113611638)

[4.2.2 Diagnostics 7](#_Toc113611639)

[4.3 Process measure 8](#_Toc113611640)

[4.3.1 Clinical decision support (CDS) 8](#_Toc113611641)

[4.3.2 Documentation 8](#_Toc113611642)

[4.3.3 Web-based e-learning module 8](#_Toc113611643)

[5. List of Analyses 9](#_Toc113611644)

[5.1 Baseline Characteristics 9](#_Toc113611645)

[5.2 Primary outcome 9](#_Toc113611646)

[5.3 Secondary outcomes 9](#_Toc113611647)

[5.4 Process measures 9](#_Toc113611648)

[6. Validation and Quality Control (QC) 10](#_Toc113611649)

[References 10](#_Toc113611650)

# List of Abbreviations

| **Abbreviation** | **Full name** |
| --- | --- |
| **ATC** | **Anatomical therapeutic chemical classification** |
| **CDI** | **Clostridium difficile infection** |
| **CDS** | **Clinical decision support** |
| **CI** | **Confidence interval** |
| **DDD** | **Defined daily dose** |
| **EPMA** | **Electronic Prescribing and Medicines Administration** |
| **GNB** | **Gram-negative bacilli** |
| **IQR** | **Interquartile range** |
| **MRSA** | **Methicillin-resistant staphylococcus aureus** |
| **QC** | **Quality Control** |
| **SD** | **Standard deviation** |
| **WHO** | **World Health Organization** |

#

# 1. Introduction

## 1.1 Background

Reducing the risk of antimicrobial resistance through appropriate antibiotic use is a major national and global challenge. The Department of Health and Social Care recommends a ‘review and revise’ approach to antibiotic prescribing in hospitals (‘Start Smart – Then Focus‘). Based on this approach, antibiotics should be started promptly for patients who have a possible bacterial infection, reviewed regularly (at least daily) within the first 72 hours of initial prescription to see if antibiotics are still needed (based on cultures and sensitivities, test results and the patient’s clinical presentation/progress) and then stopped or switched if no longer needed.

Despite national guidance, antibiotic prescribing rates are still increasing in United Kingdom (UK) hospitals. The reasons are multi-factorial: including limited real-time access to information that may influence decisions to initiate antibiotics, concerns about missing potentially serious infection (e.g. sepsis), and lack of continuity of care, which limits the opportunity for an informed review of progress and results in a reluctance to delay initiation of an/or change/discontinue an antibiotic that has already been commenced. The substantial time pressures that clinicians face compound these challenges.

This statistical analysis plan covers the quantitative analysis. Specifically, this plan does not cover the qualitative assessment data, which is covered within a separate IRAS ID: 259104), or the development and derivation of the Fidelity Index.

This document has been prepared based on the ECTU standard operating procedure, ECTU_SOP_ST_04 Statistical Analysis Plans v6.0. Moreover, the details of this statistical analysis plan are based on the ePAMS feasibility study protocol version 2.0, dated 16 August 2022.

## 1.2 Objectives

1. **[Primary objective 4 in protocol V2.0]** Determine between-patient variability in total antibiotic consumption to enable planning of the definitive cluster-randomised hybrid stepped wedge clinical trial.
2. **[Primary objective 5 in protocol V2.0]** Develop methods of collecting outcome data from ePrescribing systems prior to and following the introduction of the ePAMS+ intervention.

# 2. Statistical analysis section from the protocol

## *2.1 SAMPLE SIZE CALCULATION*

*All eligible admissions to participating wards in study sites will be included in quantitative analyses. We aim to study at least 100 admissions per ward to enable precise estimation of between-patient variability in antibiotic use (primary objective 4) and to explore feasibility of data extraction (primary objective 5) across a wide range of clinical presentations. Furthermore, inclusion of a diverse range of wards and cases will support the development of the Fidelity Index (secondary objective 4).*

## *2.2 PROPOSED ANALYSES*

*For primary objective 4, the between-patient variability in total antibiotic consumption, measured as the number of defined daily doses (DDD), will be quantified using a normal linear model to determine the components of variance, including site and ward as factors in the model. Log-transformation of data to satisfy the assumptions of the normal linear model will be considered if appropriate. Time factors, including seasonal effects and the potential impact of implementation of the ePAMS+ intervention, will also be incorporated in the model as required.*

*For primary objective 5, the measures listed will be assessed according to two criteria. First, we will determine whether it is possible to derive each outcome using the information available in the data extract from the Electronic Prescribing and Medicines Administration (EPMA). Secondly, we will summarise the measures descriptively, overall and by site and by ward, with a particular focus on the rate of missing data for each outcome.*

# 3. Overall Statistical Principles

Descriptive analysis will be conducted to present the explanatory variables by ward, study site and overall. We will present dichotomous and categorical variables as counts (percentages) in each category. Furthermore, we will present continuous variables as mean, standard deviation (SD), median, interquartile range (IQR), minimum and maximum (the latter two being reserved for variables that will not be disclosive of participants’ identities; they will not, for example, be reported for variables such as participant age). Log-transformation of continuous variables will be considered where appropriate; where this takes place the geometric mean and SD will be reported. All performed statistical tests will be two-sided with a significance level of 5%. Moreover, the two-sided 95% confidence intervals (CI) will be presented. Proportion of missing data will be recorded for all variables. The SAS statistical package (version 9.4 or later) will be used for analysis.

## 3.1 Multiplicity

As this is a feasibility study the analyses are largely descriptive; where modelling and formal inference is performed, the emphasis will be on reporting point estimates and confidence intervals, rather than hypothesis testing. There will therefore be no adjustment for multiple testing.

# 4. Outcomes

## 4.1 Primary outcome

The primary outcome is total antibiotic consumption, measured as the number of DDD per admission. It will be derived using the World Health Organization (WHO) data set on DDD by Anatomical Therapeutic Chemical Classification (ATC) code (1) which can be found in the study folder (Z:\ECTU Current Trials\1 CURRENT PROJECTS\ePAMSplus\WP2b ePAMSplus Feasibility (ECTU)\TMF\9. CRF_STATS_DATABASE_DATA MANAGEMENT), and the ATC code included in the Cerner data extracts from each site.

## 4.2 Secondary outcomes

- Length of hospital stay (days)
- Days of therapy (and intravenous therapy)

### 4.2.1 Antibiotic Prescribing

- Number of antibiotics prescribed
- Number of antibiotic courses
- Repeat courses for same indication
- Number of courses for same indication
- Switches of frequency
- Switches of dose
- Switches from intravenous to oral
- Switches from oral to intravenous
- Switches to alternative antimicrobial
- Switches from narrow to broad spectrum
- Discontinuation of therapy
- Number of courses concordant with local guidelines for antibiotic choice/duration

### 4.2.2 Diagnostics

- Resistance rates
- Susceptibility
- Acquisition of multi-drug resistant organism
- Healthcare-associated infection
- Episodes of Clostridium difficile infection (CDI)
- Episodes of methicillin- resistant Staphylococcus aureus (MRSA)
- Episodes of gram-negative bacilli (GNB)

## 4.3 Process measure

### 4.3.1 Clinical decision support (CDS)

- CDS ‘work around’
- CDS alert frequency
- CDS alert override
- Use of CDS order set
- Time to administration
- Time to active therapy (first dose)
- Spent prescribing

### 4.3.2 Documentation

- Documentation of indication
- Documentation of duration
- Documentation of stop/review
- Documentation of decision-making
- Switches from Reserve to Watch group antibiotic
- Switches from Watch to Access group antibiotic
- Adherence to clinical guidelines
- Adherence to documented sensitivity
- Appropriate dose for indication (Decision aid will include fields to record proposed site of infection (Body System) and working diagnosis (indication)).

### 4.3.3 Web-based e-learning module

- Professional discipline
- Date/time of module completion
- Time spent on learning
- Test scores

# 5. List of Analyses

## 5.1 Baseline Characteristics

We will summarise the following baseline data before and after intervention, by ward, site and overall. We will not perform any formal statistical testing.

Demographic data

- Age (years)
- Sex [Male/Female]

##

## 5.2 Primary outcome

We will estimate the between-patient variability in the primary outcome (total antibiotic consumption, measured as the number of DDD per admission). We will perform descriptive analysis of the primary outcome. Furthermore, we will report descriptive statistics before and after intervention by ward, site and overall.

The primary outcome will be analysed using a normal linear model to determine the components of variance. We will test for normality graphically using Q-Q plot. If the normality condition is violated, we will consider performing log-transformation of data if appropriate. Moreover, we will incorporate the time factors, including seasonal effect by calendar month and the potential impact of ePAMS+ intervention implementation in the model as appropriate. Determining the effectiveness of the ePAMS+ intervention is not an objective in this modelling.

## 5.3 Secondary outcomes

Descriptive summary of the secondary outcomes listed in section 4.2 will be reported in the same way as for the primary outcome.

## 5.4 Process measures

Descriptive summary of the process measures listed in section 4.3 will be reported in the same way as for the primary outcome.

# 6. Validation and Quality Control (QC)

A second statistician will do the following to validate the main statistician’s work

1. Performing the analysis and checking the results and conclusions of the primary outcome analysis.
2. Reading the statistical report to ensure it is correct and clear in its messages.

# References

1. World Health Organization. ATC/DDD Index 2022 2021, December 14 [Available from: <https://www.whocc.no/atc_ddd_index/>.
